# From laboratory to field: cross-domain few-shot learning for crop disease identification in the field

**DOI:** 10.3389/fpls.2024.1434222

**Published:** 2024-12-18

**Authors:** Sen Yang, Quan Feng, Jianhua Zhang, Wanxia Yang, Wenwei Zhou, Wenbo Yan

**Affiliations:** ^1^ College of Mechanical and Electrical Engineering, Gansu Agricultural University, Lanzhou, China; ^2^ Agricultural Information Institute, Chinese Academy of Agricultural Sciences, Beijing, China

**Keywords:** cross-domain, few-shot learning, crop diseases, recognition, multi-domain

## Abstract

Few-shot learning (FSL) methods have made remarkable progress in the field of plant disease recognition, especially in scenarios with limited available samples. However, current FSL approaches are usually limited to a restrictive setting where base classes and novel classes come from the same domain such as PlantVillage. Consequently, when the model is generalized to new domains (field disease datasets), its performance drops sharply. In this work, we revisit the cross-domain performance of existing FSL methods from both data and model perspectives, aiming to better achieve cross-domain generalization of disease by exploring inter-domain correlations. Specifically, we propose a broader cross-domain few-shot learning(CD-FSL) framework for crop disease identification that allows the classifier to generalize to previously unseen categories and domains. Within this framework, three representative CD-FSL models were developed by integrating the Brownian distance covariance (BCD) module and improving the general feature extractor, namely metric-based CD-FSL(CDFSL-BDC), optimization-based CD-FSL(CDFSL-MAML), and non-meta-learning-based CD-FSL (CDFSL-NML). To capture the impact of domain shift on model performance, six public datasets with inconsistent feature distributions between domains were selected as source domains. We provide a unified testbed to conduct extensive meta-training and meta-testing experiments on the proposed benchmarks to evaluate the generalization performance of CD-FSL in the disease domain. The results showed that the accuracy of the three CD-FSL models improved significantly as the inter-domain similarity increased. Compared with other state-of-the-art CD-FSL models, the CDFSL-BDC models had the best average performance under different domain gaps. Shifting from the pest domain to the crop disease domain, the CDFSL-BDC model achieved an accuracy of 63.95% and 80.13% in the 1-shot/5-shot setting, respectively. Furthermore, extensive evaluation on a multi-domain datasets demonstrated that multi-domain learning exhibits stronger domain transferability compared to single-domain learning when there is a large domain gap between the source and target domain. These comparative results suggest that optimizing the CD-FSL method from a data perspective is highly effective for solving disease identification tasks in field environments. This study holds promise for expanding the application of deep learning techniques in disease detection and provides a technical reference for cross-domain disease detection.

## 1 Introduction

**Figure 1:**
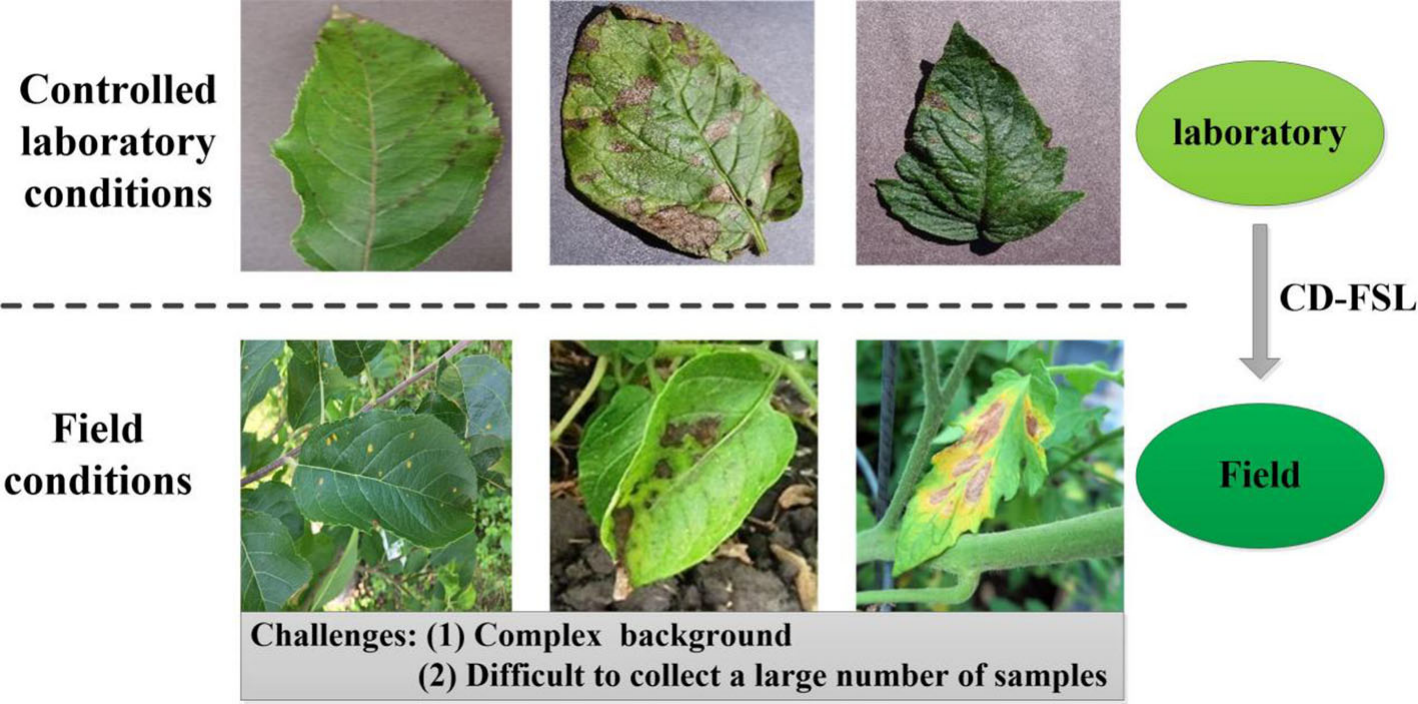
Disease images under different shooting conditions. The images in the first row are from PlantVillage and show only one diseased leaf. The second row of images was obtained in the wild. Such uncontrolled shooting conditions present challenges to the model.

Plant diseases pose a significant threat to crop growth and yield (Jadhav et al., 2021; Karthik et al., 2020). Timely diagnosis and effective control measures are crucial to mitigate disease spread and optimize agricultural productivity and quality. The characteristics of crop diseases are intricately linked to agronomic practices, climatic conditions and management levels, leading to a diverse array of pathological presentations within the same disease type. Consequently, disease recognition poses a greater challenge than traditional image classification methods. Currently, deep learning techniques have achieved great success in disease detection and identification (Dai et al., 2023; Pan et al., 2023; Rahman et al., 2020). However, the generalization ability of deep learning models depends on the size and diversity of the training datasets. In fact, collecting abundant labeled instances in field conditions, particularly for disease categories with low incidence rates, is challenging (Argüeso et al., 2020; Gui et al., 2021; Lin et al., 2022b). In addition, the collection of real-time field data under different atmospheric conditions is very time-consuming and requires a lot of expert manpower, resulting in high costs (Chouhan et al., 2020a). Many studies have achieved high accuracy in plant disease classification using deep learning models trained on PlantVillage (Alaeddine and Jihene, 2023; Alqahtani et al., 2023). Images collected in the laboratory settings are usually of good quality, with fixed background, stable illumination and obvious disease spot characteristics (**Figure 1**). However, classification networks trained in laboratory settings suffer significant performance degradation due to complex backgrounds and random imaging conditions once applied to new datasets collected from the field. This limitation severely hampers the practical utility of the model in real-world field applications. FSL methods provide an effective way to address the problem of insufficient plant disease data and improve model generalisation. However, the limitation of such methods is that they are trained and evaluated only on a single dataset (i.e., single-domain) and fail to effectively capture model properties across visual domains (cross-domain). In addition, internal and external factors such as the complexity of image backgrounds, the diversity of symptom presentations, and differences in image acquisition conditions significantly affect the generalisation ability of FSL methods. In practice, we expect that models trained on arbitrary datasets can be applied to disease datasets without the need to collect additional target training samples. Thus, the use of CD-FSL to transfer plant disease identification to more realistic and challenging field environments is important for advancing the practical deployment of intelligent diagnostic technologies.

To address the scarcity of disease samples, researchers have focused on developing simple machine learning models such as support vector machines(SVMs) (Deepa and Umarani, 2017), artificial neural networks (ANNs) and radial basis function (RBF) neural networks (Chouhan et al., 2020b) and variants. However, one of the limitations of these methods is that they require manually designed features.With the popularity of convolutional neural networks (CNN), several studies have used deep transfer learning, data augmentation, and domain adaptive techniques for disease identification. Deep transfer learning pre-train deep convolutional neural networks(DCNN) on large datasets and then fine-tune them the using disease datasets (Nigam et al., 2023; Vallabhajosyula et al., 2022). This approach is simple and easy to implement and has achieved good performance in disease recognition. For example, Chen et al (2020) used deep transfer learning for plant disease identification, yielding significant improvements in the classification performance of the DCNN model. Similarly, several studies have employed pre-trained networks such as VGGNet (Paymode and Malode, 2022), InceptionResNetV2 (Krishnamoorthy et al., 2021), MobileNetV2 (Hassan et al., 2021), Vision Transformer (ViT) (Xu et al., 2022), and EfficientNet (Atila et al., 2021)for crop disease identification. Although transfer learning is effective in scenarios facing insufficient samples, this method still relies on medium-sized databases during the fine-tuning stage. Therefore, its accuracy diminishes when faced with only a few to dozens of samples. Moreover, most existing disease recognition method depend on publicly available datasets, such as PlantVillage, and samples collected in the laboratory settings. Images obtained in field settings encompass a diverse range of backgrounds and symptom features (Barbedo, 2018). Some studies have segmented leaves or spots from complex backgrounds to improve disease recognition accuracy (Chouhan et al., 2021). Automatic recognition of plant disease in field environment remains a formidable challenge, particularly when disease datasets are scarce. To diversify disease datasets and combat the challenge of small dataset sizes, many studies have employed data enhancement techniques to employed overfitting in DCNNs. Common methods such as image rotation, scaling, shifting, and color transformations were used to expand capacity of disease datasets (Nagaraju et al., 2022; Enkvetchakul and Surinta, 2022). However,these techniques merely apply algorithms to transform raw images into augmented ones without fundamentally enhancing model generalization. Therefore, efficient generative adversarial networks (GANs) have been utilized for disease data enhancement to generate a diverse array of new disease samples (Cap et al., 2020). For example, Haruna et al. (2023) used Style-Generative Adversarial Network Adaptive Discriminator Augmentation (SG2-ADA) and Laplace filter variance to synthesise images of rice leaf diseases. Furthermore, variants of GAN architecture (Liu et al., 2020) and StyleGAN (Pandian et al., 2019) have also been introduced into deep learning frameworks to improve the accuracy of identifying diseased plant leaves. The distribution of disease data typically follows a long-tailed shape, with a small fraction of categories having abundant data and the majority having limited data. While data augmentation methods have made significant progress in addressing data limitations and balancing the distribution of disease data, the model performance remains limited by the size of the benchmark datasets. Currently, public disease datasets or self-built datasets are obtained through different sensing platforms, lighting conditions, and background settings. Discrepancies in data distribution originating from diverse sources often result in a decline in model performance. Domain-adaptive techniques aim to mitigate these differences by minimizing the disparities in probability distributions between source and target domains, thereby producing embedded datasets that share a common subspace. Zhao et al. (2023) proposed a domain-adaptive self-supervised comparative learning for plant disease identification. This approach demonstrates high accuracy even in scenarios with limited training data and cluttered unlabeled data. However, it requires that the training and test sets have matching label types. Given the limitations of the aforementioned methods, there is an urgent need to explore disease classification methods that can be applied to small datasets.

FSL represents a pioneering paradigm centered on task-driven methodologies, allowing models to adapt to novel tasks with limited labeled data (Sun et al., 2023). In general, the standard setup for learning few-shot classifiers consists of two phases: 1) training the model on the datasets from the source domain, and 2) testing the novel task using a small set of supports in the target domain. Currently, meta-learning plays a crucial role in FSL, addressing the challenge of limited samples in specific domains such as disease diagnosis (Chen et al., 2021; Argüeso et al., 2020).Various meta-learning techniques, including optimization-based, model-based, and metric-based methods, have been introduced (Parnami and Lee, 2022). Lin et al. (2022a) proposed a solution to the problem of limited feature extraction in few-shot learning by using cascaded multi-scale feature fusion and a channel attention module. Li and Yang, 2021 investigated the impact of domain transfer and meta-learning parameters (e.g., N-way, K-shot) on the performance of FSL recognition methods using a meta-learning baseline. Similarly, Yan et al. (2023) addressed the issue of low accuracy for a single metric model by combining three metric networks, namely prototypical network, matching network, and DeepEMD, to create an enhanced FSL network. Although FSL methods have shown advantages in overcoming data shortages, two challenging issues remain: (1) The datasets for both meta-training and meta-testing of existing FSL come from the same domain or dataset. For example, previous studies have scaled PlantVillage datasets or self-constructed datasets into base and new classes. They perform poorly on the more challenging cross-domain FSL task, where test data come from previously unseen domains. (2) The performance of FSL models trained on a specific dataset (controlled environment) is greatly reduced when generalised to new datasets or environments.

In this work, we attempt take advantage of cross-domain FSLs to address the problem of inter-domain knowledge transfer and generalisation, and to improve the accuracy of disease recognition with small datasets. This method considers learning models from one or multiple domains and extends them to unknown domains with fewer samples. This learning paradigm introduces additional challenges, requiring the use of limited datasets to learn new tasks and the targeted transfer of prior knowledge from visible domains to new tasks. Current mainstream methods primarily focus on improving the network structure by incorporating task-specific learnable modules and feature transformation layers to adapt to new tasks (Li et al., 2021a; Li et al., 2022). These methods emphasize the network structure improvement while overlooking the influence of the data distribution space on model generalization. For example, Convolutional Autoencoder (CAE), Feature Attention Module (Rezaei et al., 2024), and Attention Generating Adversarial Networks combined with FSL are used for plant disease recognition in low data scenarios. Mu et al. (2024) proposed a small-sample disease recognition algorithm for supervised comparative learning, and the model achieved an accuracy of 79.51% in a cross-scene potato disease recognition task. Garg (2023) developed a lightweight few shot model for plant leaf disease classification based on aggregated loss function with MobileNetV2. Additionally, existing CD-FSL methods have already established standard benchmarks using shared meta-data. These datasets share the common characteristic of coarse granularity, meaning a higher degree of class visual distinctiveness within the datasets. Currently, it is unclear how the CD-FSL method performs on challenging disease datasets.With this background, we reexamine the cross-domain problem from a data perspective and develop an economical, efficient, and broader applicable cross-domain disease recognition framework for small-sample. Unlike previous studies that rely solely on network improvements, the motivation of this work is to introduce appropriate source domain datasets and optimize network structures to enhance the accuracy of CD-FSL models for crop disease diagnosis in field environments. To achieve this goal, the following research objectives are proposed:

1. We collected disease images from public resources and real agricultural fields, and established a crop disease dataset in a field environment. This dataset contains 9 crops covering 43 different disease types. We hope that this dataset will be helpful for future research on plant disease diagnosis.
2. We propose a broader CD-FSL framework to address the shortage of disease samples and verify the adaptability of different cross-domain FSL methods to disease tasks in field scenarios. This work provides a new perspective and approach to disease identification.
3. We thoroughly investigate how inter-domain variability and the diversity of source domain data affect the accuracy of crop disease identification. The experimental results can serve as a benchmark and reference for subsequent cross-domain FSL in the field of disease identification.

## 2 Materials and methods

### 2.1 Image collection

**Figure 2:**
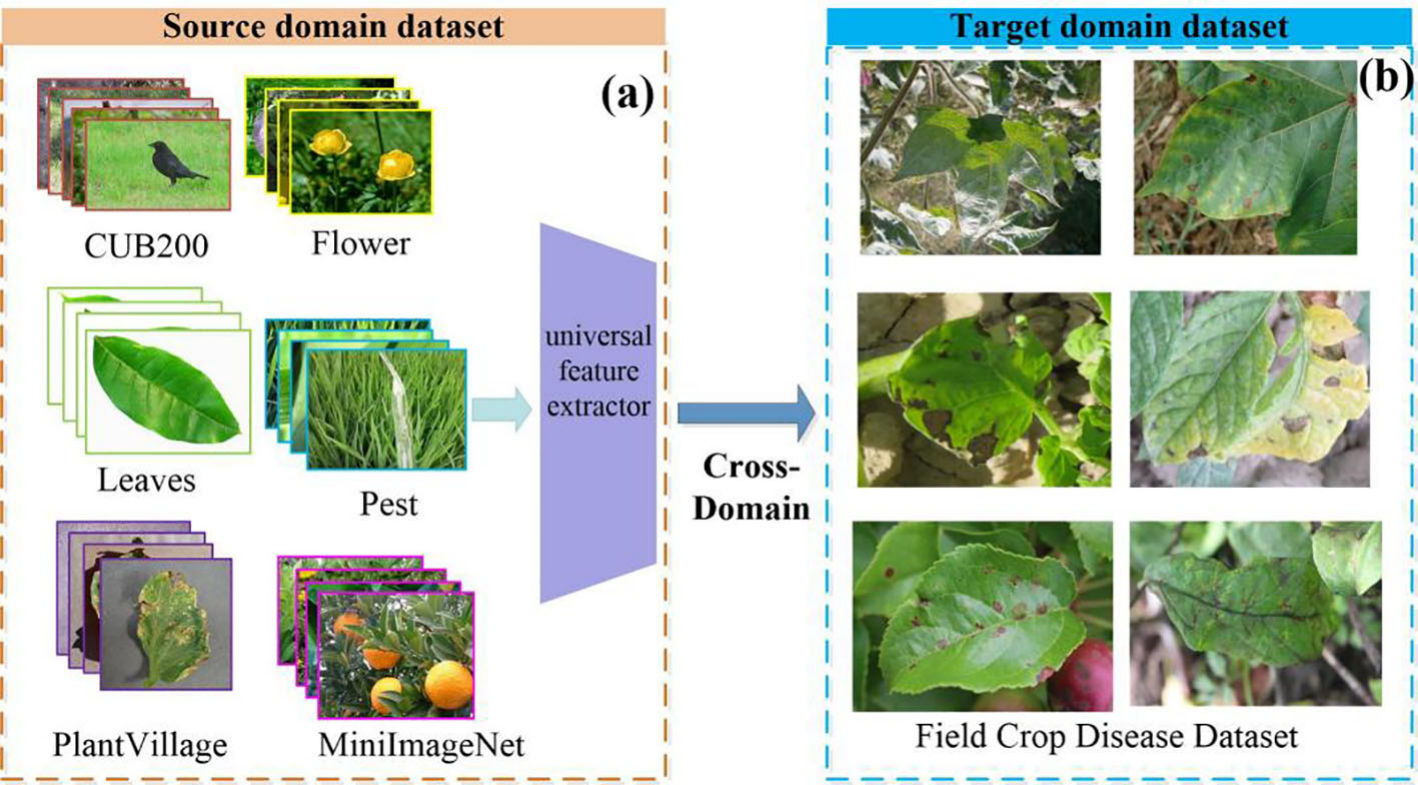
Example source **(A)** and target **(B)** domain datasets. The source domain is a publicly available dataset commonly used for image classification tasks. It is used to train general feature extractors. The CUB200 dataset is rich in visual detail and is designed for fine-grained image classification tasks. The Leaves dataset covers a wide range of species of healthy plant leaves with a single background. The Flower dataset has a rich variety of colours, shapes and texture features. In contrast, the Pest dataset presents images with complex backgrounds, exhibiting considerable inter-class variations. The MiniImageNet dataset has large differences in visual features between classes. The target domain involves a plant disease dataset, collected under field conditions, where images are subject to intricate backgrounds and uneven lighting, posing additional challenges for accurate classification.

The dataset contains the source domain (base class) and the target domain data (novel class). Seven public datasets were selected as source domains, as shown in **Figure 2**. These datasets have been widely used in CD-FSL research and have good benchmarking properties that facilitate comparisons with existing studies. In addition, these datasets cover a wide range of tasks from fine-grained classification (e.g., Cars, CUB200) to domain-related (e.g., PlantVillage and Pests), and are able to provide a representative distribution of data. Among them, the Leaves dataset consists of the Kaggle dataset and the Flavia dataset, while all other source domain datasets are standard public datasets. CUB200(200 classes) (Wah et al., 2011) and Cars(100 classes) (Krause et al., 2013) belong to two broader datasets that differ significantly from disease images in terms of semantic content, context and colour. While the Cars and CUB200 data may not seem relevant to the agricultural disease domain on the surface, the fine-grained classification capabilities and complex contexts in these two datasets can be migrated to agricultural disease scenarios. The Flower (102 classes) (Nilsback and Zisserman, 2006) and Leaves(208 classes) (Azlah et al., 2019) datasets involve morphological features of plants, flowers and leaves that are similar to the disease dataset, and the diversity of the field scenes is also similar to the field disease images as well. These features contribute to the model’s ability to extract cross-domain representations that are relevant to disease identification. The PlantVillage (Hughes and Salathé, 2015) dataset consists of 54,303 leaf images, covering 38 diseases across 14 crops. Although this disease dataset has a large sample size, all images were collected under controlled conditions and therefore cannot be used as a substitute for disease recognition tasks in the field. The backgrounds of the Pest(102 classes) (Wu et al., 2019) dataset are mostly leaves, stems or flowers in field environments, which are consistent with the scenes of disease occurrence. In addition, the collection environment of the Pest dataset has similar light, angle and resolution conditions as the field disease images. The coarse-grained dataset MiniImageNet (Ravi and Larochelle, 2016) (100 classes) is often used for network pre-training. To ensure the breadth and diversity of cross-domain learning, the source domain dataset contains data with various inter-domain variations. It covers datasets highly similar to plant disease distribution, such as PlantVillage and Pest, as well as datasets less relevant to the disease domain, like Cars, CUB200, and MiniImageNet.

The target domain data are images of crop diseases taken from field scenes. The database was obtained through three ways: self-constructed, internet collection and public data download. The dataset is drawn from different regions and provides comprehensive coverage of the universality and diversity in different agricultural environments. It includes nine crop diseases, namely apple, potato, maize, cotton, cucumber, grape, rice, tomato, and wheat, amounting to a total of 43 disease categories. The detailed distribution of disease category is shown in **Table 1**. In this study, this dataset was named the Field Crop Disease Dataset (FCDD). In **Figure 2B**, examples of disease images are displayed. These images were taken in field scenes, which present a complex background with diverse leaf angles and uneven lighting. Additionally, the images contain confounding factors such as light spots, shadows, and insect eyes. The distribution of the FCDD dataset is consistent with the characteristics of the field operating environment, providing a benchmark database for disease identification research transitioning from the laboratory to the field. Compared to PlantVillage and Pest, the main advantage of the FCDD dataset is its realistic simulation of real agricultural environments, allowing a more comprehensive test of the model’s performance and migration capabilities in complex, dynamic and diverse scenarios. This type of data is closer to the actual needs of agricultural disease monitoring and provides a higher reference value for model use in the field.

**Table 1:** The detailed distribution of the FCDD dataset.

Crop	Number of categories	Categories
Apple (Thapa et al., 2020)	4	Apple scab, apple rust, apple multiple, healthy
Potato	3	Early blight, late blight, healthy
Corn (Qian et al., 2022)	4	Gray leaf spot, corn leaf blight, corn rust, healthy
Cotton	7	Areolate mildew, bacterial blight, cercospora leaf spot, curl virus, target spot, verticillium wilt, healthy
Cucumber	4	Powdery mildew, downy mildew, angular leaf spot, healthy
Grape	4	Black rot, black measles, leaf blight, healthy
Rice (Sethy et al., 2020)	4	Bacterail blight, blast, brown spot, tungro
Tomato	10	Bacterial spot, target spot, early blight, late blight, leaf mold, mosaic virus, septoria spot, spider mites,yellow virus, healthy
Wheat	3	Strip rust, septoria, healthy

### 2.2 Problem description of cross-domain few-shot learning

**Figure 3:**
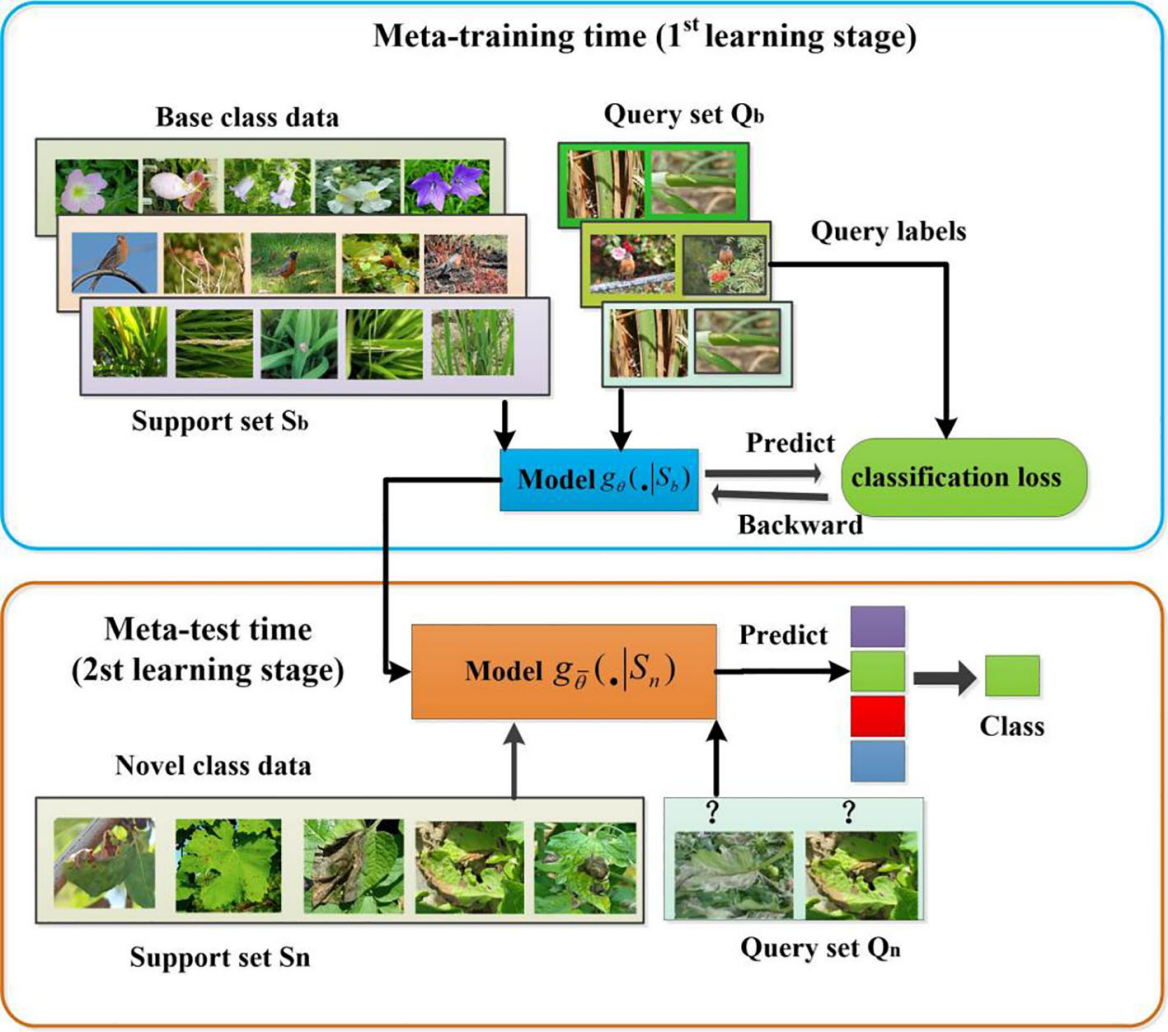
Framework of the proposed CD-FSL model for field crop disease recognition.

The goal of cross-domain few-shot learning is to enable models to generalize not only across a single data distribution, but also to previously unseen data distributions. In CD-FSL, the model is trained on source domain dataset $D_{s}^{seen}$ , and then tested on target domain dataset $D_{t}^{useen}$ . Typically, the training and testing of CD-FSL is performed in an episode-based way. Each task τ=(S,Q) is a small dataset consisting of a support set S and a query set Q . The support set *S* and the query set *Q* are composed of $K_{s}$ and $K_{q}$ samples respectively, taken from each category. Since the support set has *N* categories and each category has *K* samples, this type of data composition is called the ‘N-way K-shot’ problem. CD-FSL is a learning approach that emphasizes the acquisition of prior knowledge from past experiences to enhance the learning of new tasks. It consists of two primary components: meta-training and meta-testing, as shown in **Figure 3**. In meta-training, a large number of N-way-K-shot source tasks are created using source domain data. The model has to learn from a support set $S_{b}={\left\{x_{i},y_{i}\right\}}_{i=1}^{N\times K}$ , and is then evaluated on a query set $Q_{b}={\left\{x_{j},y_{j}\right\}}_{j=1}^{N\times F}$ . Here $x_{i},x_{j}$ denotes the samples of the support set and the query set, $y_{i},y_{j}$ denotes the labels of the corresponding samples, *F* denotes the number of samples of each class in the query set, usually *F* takes 15. During meta-testing, the model parameters are re-tuned using the novel class query set $S_{n}$ to generate a new classifier $g_{\bar{\theta }}{(}_{•}|S_{n})$ . The classifier is able to quickly adapt to the CD-FSL disease task in the target domain with only a few samples, as it has learned the prior knowledge θ of the N-way K-shot task in the source domain.

Assuming that the joint distribution of tasks τ is P(τ) , then the joint distributions of the tasks sampled from the source and target domains are $P_{s}(\tau )$ and $P_{t}(\tau )$ , respectively. The model acquires knowledge on independently sampled tasks and then generalizes the model to new tasks. Both the meta-learning and meta-testing tasks of the generic FSL model come from the same sub-dataset, so the spatial distribution of data in the source and target domains are similar, i.e., $P_{s}(\tau )$ = $P_{t}(\tau )$ . In cross-domain learning, the test data (target domain) is usually sampled from unknown or unseen domains. For example, in this study, the target domain domain is a specific FCDD dataset, while the source domain data is any data source in the $D_{s}^{seen}$ . There is usually be a large difference between the data distributions of the source and target domains, so the task distribution of a cross-domain FSL usually satisfies $P_{s}(\tau )$ ≠ $P_{t}(\tau )$ . This domain gap poses new challenges for cross-domain generalisation of models.

According to the number of sub-datasets in the source domain, CD-FSL is categorized into single-domain learning and multi-domain learning. In single-domain learning, both meta-training and meta-test examples are sampled from only one dataset. In multi-domain learning, (S,Q) are sampled from multiple datasets. The objective of meta-learning is to enable the classifier to achieve optimal performance in each task $\tau_{i}$ . The specific optimization process is described below:

1. Train the classifier $g_{\theta }{(}_{•}|S_{b})$ using the support set $S_{b}$ ;
2. Calculate the classification score $p_{m}=g_{\theta }{(}_{•}|x_{j}^{Q_{b}})$ for each sample $x_{j}^{Q_{b}}$ in the query set $Q_{b}$ ;
3. Calculate the loss $L(g_{\theta }{(}_{•}|S_{b}),Q_{b})$ based on the query set labels and classification scores, and optimise the classifier parameters θ .

In the training or meta-training stage, the model is trained (or meta-trained) on the source domain data to learn generic feature extractors. This process models aim to capture shared features that are useful for multiple tasks. In the meta-testing stage, multiple tasks N-way K shot were extracted from the target domain, each consisting of a support set *S*_n_ and a query set *Q_n_*. Before each use of the query set *Q_n_* to evaluate the performance of the model, we use the support set *S_n_* of the tasks in the target domain to re-fine tune the model network parameters. By using support sets in each task, the model is able to dynamically adjust its parameters to better fit the features of the target domain. This fine-tuning allows the model to capture patterns specific to the target domain, thus improving its performance on the query set *Q_n_*.

### 2.3 Building CD-FSL model for disease identification

#### 2.3.1 Universal feature extractor

**Figure 4:**
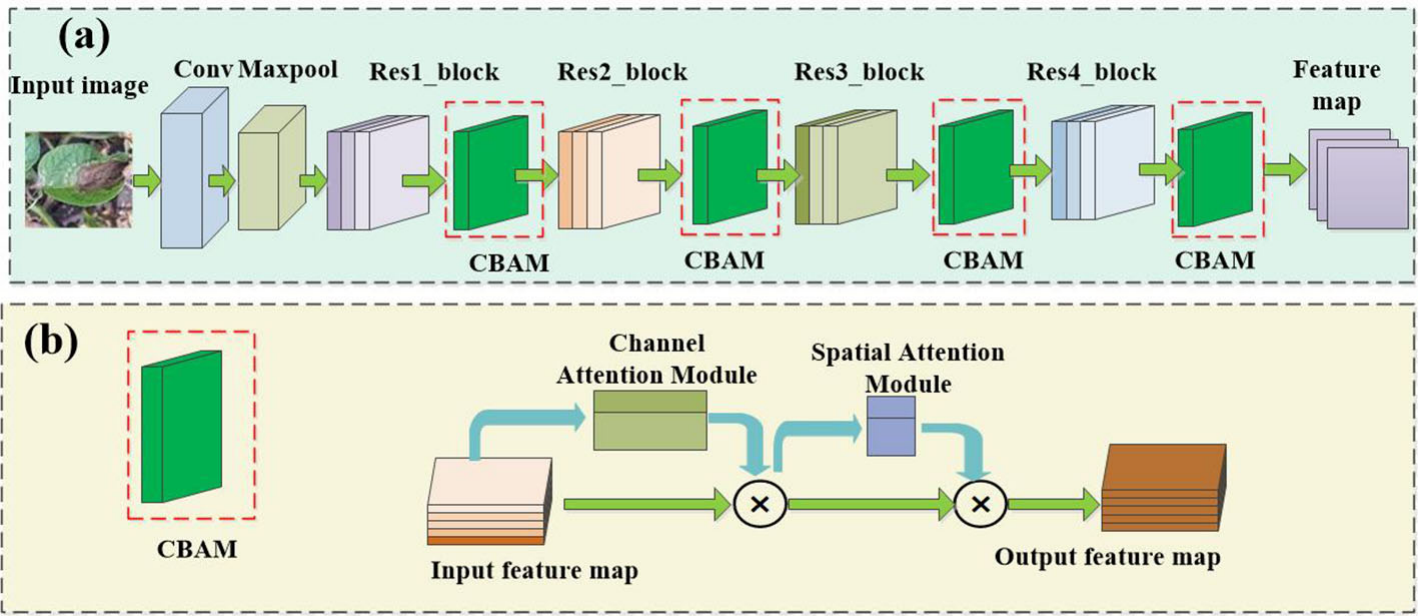
Network structure of universal feature extractor. **(A)** ResNet 18, **(B)** CBAM.

Extracting effective general feature representations from any given source domain is essential for the cross-domain generalization of CD-FSL. Previous research has shown that most FSL methods directly adopt ResNet12 or ResNet18 backbone networks as universal feature extractors. However, these methods use source and target domains from the same data distribution. In the presence of a domain gap, a good task-agnostic feature extractor is expected to produce feature representations of previously unseen tasks and domains. In this study, we propose an augmented ResNet architecture by incorporating the convolutional block attention module (CBAM), as shown in **Figure 4A**. CBAM consists of two key parts (**Figure 4B**): channel attention and spatial attention. This module adjusts the weights of different channels and different spatial positions to enable the model to better focus on important channel-wise and spatial location information. Specifically, the CBAM module is added after each convolutional block of the ResNet-18 universal feature extractor to enhance the network’s ability to extract universal features.

#### 2.3.2 CD-FSL model with BDC module

**Figure 5:**
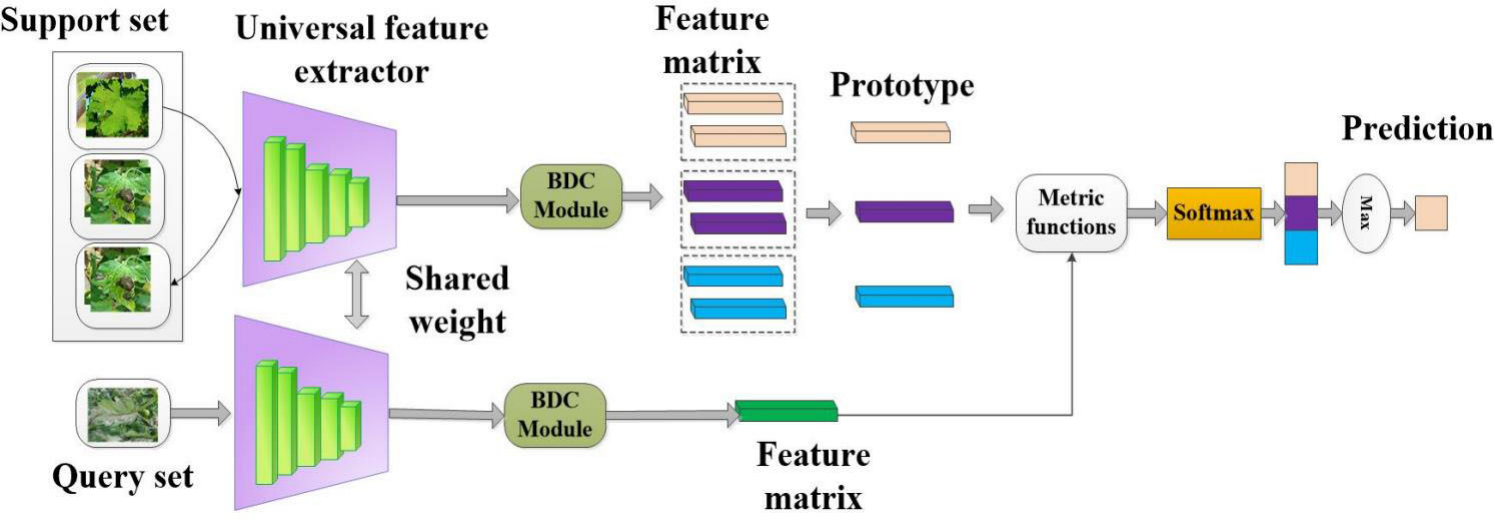
Cross-domain few-shot classification algorithm with metric learning to solve the crop disease identification task. The figure shows a specific example of a 3-way 2-shot classification task.

In this study, we designed a cross-domain FSL model based on the ProtoNet network. This framework consists of a universal feature extractor, a feature embedding module, and a similarity measurement and classifier, as shown in **Figure 5**. This model is named CDFSL-BDC in this study. To generate efficient generic feature representations, the shared feature extractor for the support set and query set adopts the proposed ResNet-18+CBAM model. Typically, the high-dimensional features extracted from the support set and query set by the shared feature extractor are often nonlinear. This complexity makes it difficult to obtain effective low-dimensional feature embeddings for the metrics module, potentially reducing its ability to generalise across domains. To effectively measure the similarity between the support image *S* and the query image *Q* and improve the cross-domain capability of CD-FSL, the BDC module is embedded at the back end of the universal feature extractor (Xie et al., 2022). BDC can evaluate the dependency relationship between two random variables by measuring the Euclidean distance between the joint feature function and the marginal product of embedded features. This module mainly involves standard matrix operations, and the specific calculation process is as follows:

1. Reduce the dimensionality of the high-dimensional feature maps outputted by the universal feature extractor using a 1×1 convolutional layer, and then reshape them into $\text{X}\in {\text{R}}^{wh\times d}$ , where *w*, *h*, and *d* are the width, height, and number of channels of the feature maps.
2. Compute the squared Euclidean distance matrix $\tilde{\text{A}}$ and the Euclidean distance matrix $\hat{\text{A}}$ for the input *X*.
3. Subtract the row, column, and overall mean from matrix $\tilde{\text{A}}$ to obtain the BDC matrix $\tilde{\text{A}}$ .

In this process, the extraction of the BDC matrix can be considered as a non-parametric pooling operation. Unlike these traditional parameterized pooling operations, BDC does not involve a learning process for the parameters. Rather, it is a non-parametric method based on distance and covariance, employed for secondary feature representation extraction from images. During the meta-training stage, the source domain dataset is sampled into a large number of tasks for model training. The support set in each task is used to enable the model to build a classification pattern, while the query set is used to verify the effectiveness of the pattern. Specifically, *K* images of each category in the support set are fed to the universal feature extractor and BDC module to obtain the BDC feature matrix for each category. Then, the average of the *K* BDC matrices of category c is calculated as the prototype of that category. The specific expression is as follows:


(1) $$v_{c}=\frac{1}{K}\sum_{(x_{i},y_{i})\in S_{c}}{\text{A}}_{\theta }(x_{i})$$

Where $S_{c}$ denotes a subset of category c , $\left(x_{i},y_{i}\right)$ denotes a sample taken from $S_{c}$ and the corresponding label.

The similarity between a sample from the query set and all prototypes in the embedding space is determined by mapping the feature embeddings. Any one of the metric functions, such as Euclidean distance, cosine distance, inner product and Earth Mover’s Distance (EMD), can be employed to compute the similarity between embeddings.

The objective of meta-training is to update the parameters of the feature extractor and the metric function based on the average error computed on a query set of multiple N-way-K-shot episodes. Therefore, the cross-entropy loss is employed to update the parameters of the network. For a few-shot episode, the loss function of the proposed method is as follows:


(2) $$L(\theta )=-\log\frac{\exp(-d({\text{A}}_{\theta }(x_{q}),v_{c}))}{\sum_{c^{'}}\exp(-d({\text{A}}_{\theta }(x_{q}),v_{c'})}$$

where $x_{q}$ is the query set sample, $c^{'}$ represents all classes in *S*, $v_{c'}$ represents all prototypes in *S*, and d(.) represents the Euclidean distance.

During the meta-testing stage, the feature extractor and the BDC module are applied to the samples within the support set of the target domain (crop disease data) to obtain the feature embeddings for each sample. Subsequently, the distance between the samples in the query set and the prototypes of each category is computed. Finally, the test samples are assigned to the category represented by the prototype with the closest distance.

This work also applies FSL methods to a non-meta-learning framework for cross-domain disease recognition. We refer to this method as CDFSL-NML. In this method, a cross-scene spatial embedding is trained on the source domain, followed by the transfer of knowledge, features, or model parameters from the source domain to the disease target domain. Unlike the CDFSL-BDC approach, CDFSL-NML adopts a non-meta-learning training mode, where all class samples in the source domain are used as the training set to optimize the model parameters. During the testing phase, the target domain still follows the N-way K-shot benchmark. Each task consists of a support set *S_n_* and a query set *Q_n_*. The support set contains samples used for training and fine-tuning the model, while the query set is used is employed to evaluate the performance of CDFSL-NML on unseen samples.

**Figure 6:**
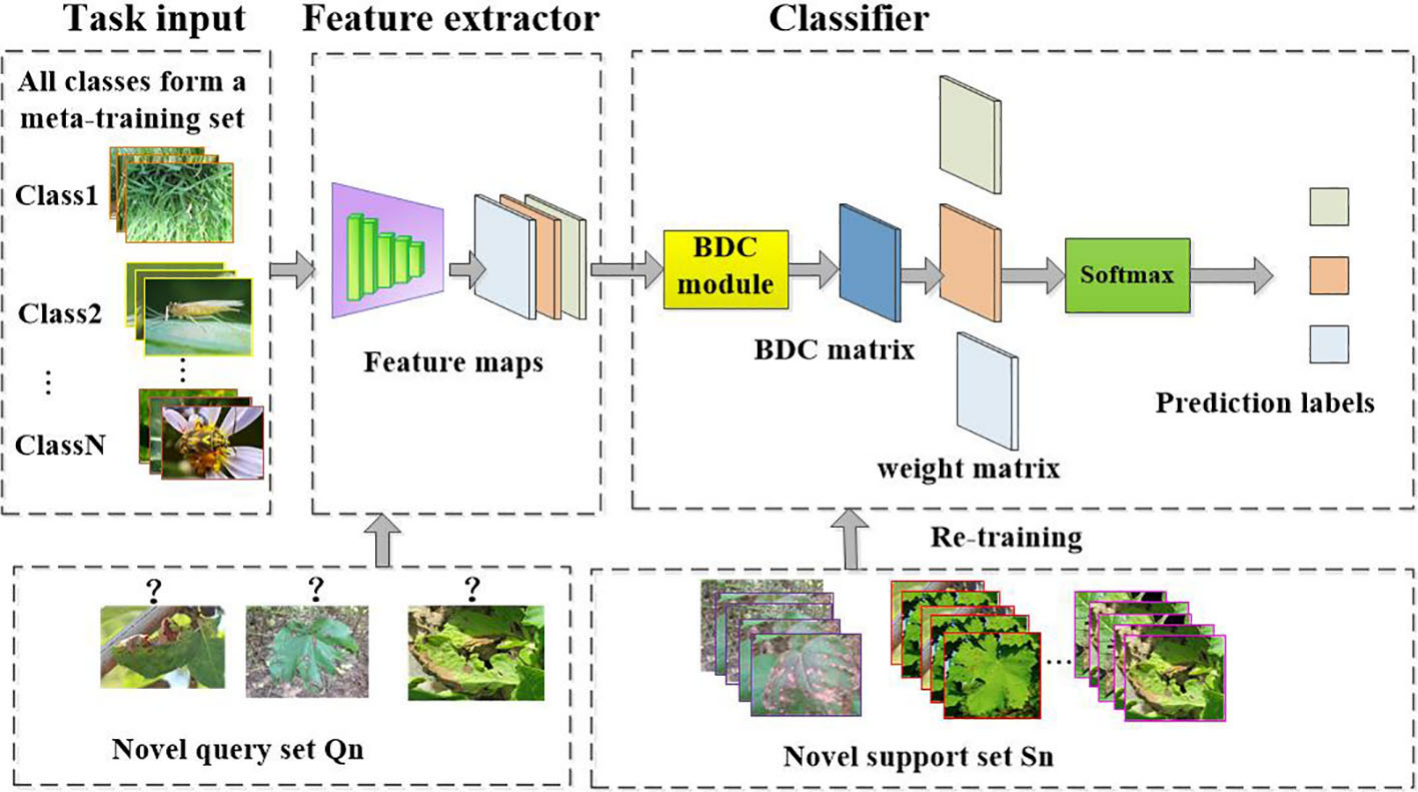
Cross-domain few-shot classification algorithm based on non-meta learning to solve the crop disease identification task. Unlike CDFSL-BDC, CDFSL-NML uses non-episodic training on source domain data.

The framework of CDFSL-NML is illustrated in **Figure 6**. The process includes the following steps:

1. Train a cross-domain universal classifier using the entire data from the source domain.
2. Then retrain a new linear classifier using the support set *S_n_* of the new task.
3. Finally, evaluate the adaptability of the CDFSL-NML model on disease classification tasks through the query set *Q_n_*.

Given the source data training set or the target domain query set, we train the feature extraction network and the classifier using the cross-entropy loss between the predicted and true labels. The loss function is expressed as (Xie et al., 2022):


(3) $$\underset{\theta ,\;{\text{W}}_{k}}{\text{arg min}}-\sum_{({\text{z}}_{j},y_{j})\in C^{\text{train}}}\log\frac{\exp(\lambda \;tr({\text{A}}_{\theta }{\left({\text{z}}_{j}\right)}^{T}{\text{W}}_{yj}))}{\sum_{k}\exp(\lambda \;tr({\text{A}}_{\theta }{\left({\text{z}}_{j}\right)}^{T}{\text{W}}_{k}))}$$

Where ${\text{W}}_{k}$ is the *k-th* weight matrix, λ is a learnable scaling parameter, and tr(·) is the matrix trajectory. T represents the transpose of the matrix, $\left(z_{j},y_{i}\right)$ represents the input image and label pair of the model, and ${\text{A}}_{\text{θ}}({\text{z}}_{\text{j}})$ represents the BDC matrix generated by the input network of image $z_{j}$ .

#### 2.3.3 CD-FSL model based on optimization meta-learning

**Figure 7:**
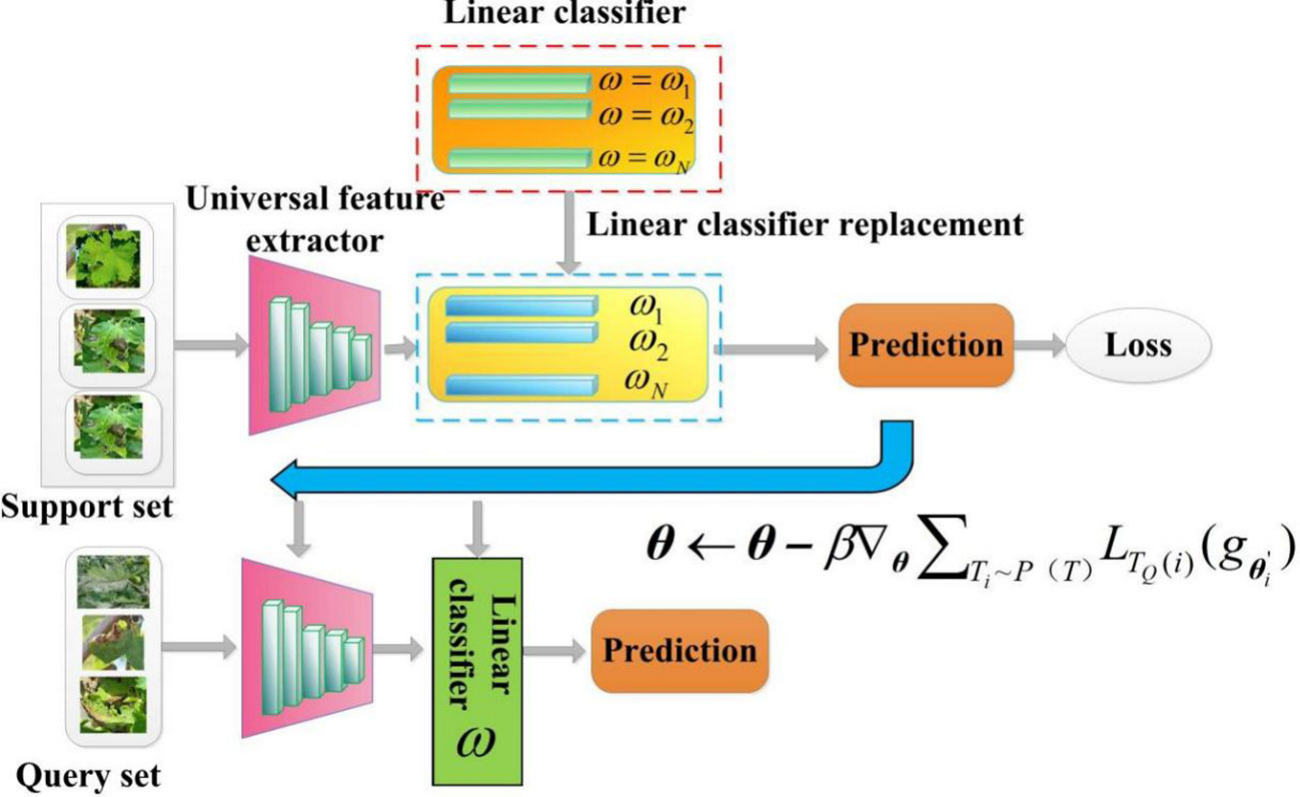
Cross-domain few-shot classification algorithm with MAML. The goal of CDFSL-MAML is to optimise a parameter θ on the source domain so that it can be quickly adapted to the disease task. During the meta-training stage, both the support set and the query set are sampled from the source domain. In the meta-testing stage, the support set and query set are sampled from the target domain FCDD.

Model-agnostic meta-learning (MAML) is a important branch of FSL methods, based on optimization meta-learning techniques (Ye and Chao, 2021). Due to its model-independent nature, any network can be used as a learner in its framework. In this study, we also employed the MAML algorithm and the meta-learner to design an optimization-based CD-FSL model (CDFSL-MAML). The meta-learner consists of a ResNet12 feature extractor and a linear layer. To avoid overfitting, the universal feature extractor of this method adopts a shallow Resnet-12 network. **Figure 7** illustrates the disease recognition process based on CDFSL-MAML. The meta-learner consists of a ResNet12 feature extractor and a linear layer. During the meta-training stage, MAML requires initializing the parameters of the linear classifiers. For N-way K-shot tasks, standard MAML initializes N-way different classifiers $\{{\omega_{c}\}}_{c=1}^{N}$ . However, the randomness of the label assignment permutations during meta-testing can lead to significant differences in accuracy. To reduce the sensitivity of MAML to label permutations in cross-domain learning, consider initializing the linear classifier with N identical weight vectors ω .

The CDFSL-MAML formulation is a two-layer optimisation problem consisting of an inner-loop optimisation and an outer-loop optimisation. For the set of input samples *x*, the meta-learner $g_{\theta }(x)$ is defined as:


(4) $$g_{\theta }(x)=\arg\max\omega_{c\in N}^{T}f_{\phi }(x)$$

Where $f_{\phi }$ is the feature extractor, ω is the weight vectors of the linear classification head, and θ is the parameter of the meta-learner, θ={ω,ϕ} .

The optimisation process of CDFSL-MAML is as follows:

1. Initialize model parameters θ , and extract a collection of tasks (episodes) to update model parameters θ to $\theta^{\prime }$ .

(5) $${\text{θ}}^{\prime }=\text{θ}-\alpha \nabla_{\text{θ}}L_{T_{S}(i)}(g_{\theta })$$

2. Calculate the sum of the losses of the query set in the training task as the overall meta-loss and then update θ .

(6) $$\text{θ}\leftarrow \text{θ}-\beta \nabla_{\text{θ}}\sum_{T_{i}∼P(T)}L_{T_{Q}(i)}(g_{{\text{θ}}_{i}^{'}})$$

Where β is the meta-step size, Task $T_{i}$ is drawn from the task distribution P(τ) , $T_{S}(i)$ is the support set of the *i-th* task, and α is the learning rate.

In the meta-training stage, the meta-learner acquires good initial parameters by optimizing them with the source domain dataset. This process allows the model to easily adapt to new tasks. In the meta-testing stage, the model performs gradient descent on the support set of the target domain, calculates the gradient by backpropagation, and updates the model parameters to adapt to the current task. It is important to note that since the meta-learner has already learned excellent initial parameters during meta-training, it can quickly optimize for new disease tasks using only a small amount of data for gradient descent. Finally, different query sets are selected for multi-task evaluation to comprehensively assess the model’s ability to learn across different domains.

### 2.4 Measures of domain similarity

The raw data from both the source and target domains are encoded into the feature space using a feature extractor. Each domain is characterized by a feature space mean, reflecting the average position of all domain samples within the feature space. Cosine similarity serves as a metric to evaluate the similarity between two vectors within the feature space, with larger values indicating heightened similarity. In this study, cosine similarity is employed to assess the similarity between the feature space means of the source domain and target domain, determining their inter-domain correlations in feature distribution. The formula is as follows:


(7) $$d(X,Y)=\frac{\text{Avg(}f_{\phi }(D_{S}))\cdot \text{Avg}(f_{\phi }(D_{t}))}{\left|\text{Avg}(f_{\phi }(D_{S}))\right|\left|\text{Avg}(f_{\phi }(D_{t}))\right|}$$

Where $D_{S}$ denotes the sample of the source domain, $D_{t}$ denotes the sample of the target domain, Avg(.) denotes the mean operation of the feature space vector, *X* denotes the mean vector of the source domain, and *Y* denotes the mean vector of the target domain. The closer d(X,Y) is to 1, the higher the similarity between different domains, otherwise the higher the difference of the data distribution between different domains.

In addition, to ensure the accuracy of the similarity metric, Euclidean distance was also used to measure the similarity between the source and target datasets and the metric was compared with Cosine similarity.

### 2.5 Evaluation of indicators

We validate the proposed method following the standard CD-FSC evaluation scheme (Li et al., 2022a). In each target domain, we randomly sample 600 N-way K-shot 15 query tasks and calculate the average accuracy of these sampled tasks. The calculation formula is as follows:


(8) $$Acc_{av}=\frac{1}{t}\sum_{k=1}^{t}Acc_{k}$$

where *t* is the number of extraction tasks, *t=*600, $Acc_{\text{k}}$ is the classification accuracy of the *k-th* task.

## 3 Results

### 3.1 Experimental details

The datasets involved in this study include disease and other multi-domain data. The target domain is FCDD, while the source domain is 9 public datasets. These datasets were used to train the model with the aim of enabling it to learn universal features that can be applied to the disease domain. Three methods, namely CDFSL-BDC, CDFSL-MAML, and CDFSL-NML, are developed based on cross-domain few-shot learning frameworks. These methods use ResNet-18+CBAM as the backbone network for feature extraction. During the meta-training stage, CDFSL-BDC and CDFSL-MAML adopt an N-way, K-shot task training, while CDFSL-NML uses a non-episodic training. For the meta-testing stage, all three methods are tested using 5-way-1shot and 5-way-5shot tasks. In the experiments, the query set of each category comprises 15 images, and the average accuracy is evaluated based on 600 randomly sampled tasks. The pre-training of all CD-FSLs was performed on MiniImageNet. The hyperparameters of the pre-training stage were set to batch size 64, epoch 400, learning rate 5×10^-2^ and dropout rate 0.6. The hyperparameters of the meta-training stage (training) stage were set to batch size 64, epoch 60 and learning rate 1×10^-3^.

### 3.2 Cross-domain performance evaluation of CD-FSL under different source domains

**Figure 8:**
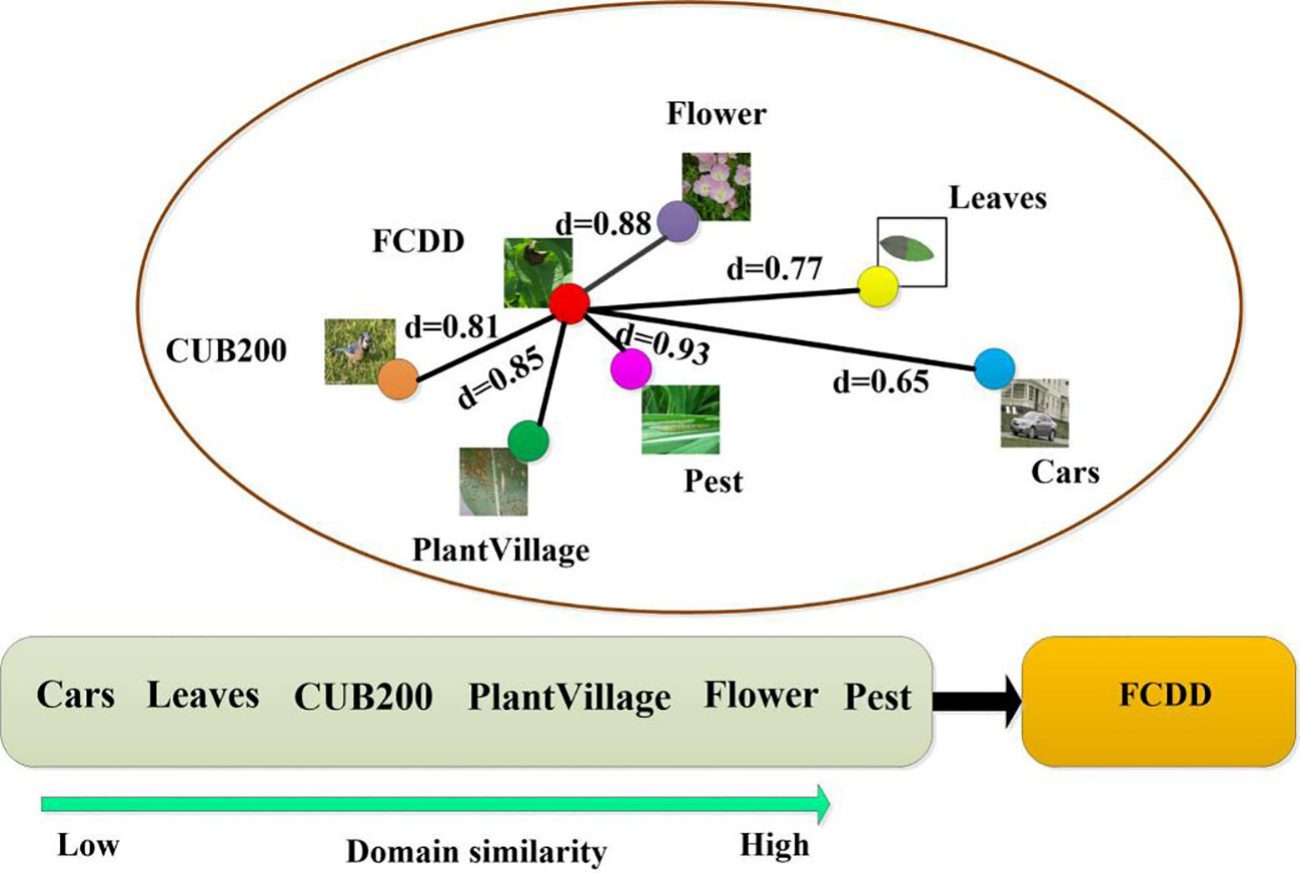
Space of similarity distributions between source and target domains based on.

The inter-domain similarity between the source and target domains was quantitatively analysed using Cosine similarity and Euclidean distance (**Table 2**; **Figure 8**). Overall, the two similarity measure functions show the same trend in 6 different source domain data sets. The Cars dataset has the lowest domain similarity (*d*=0.65/17.13) and demonstrates a significant deviation in semantic content from the FCDD. The Leaves and CUB200 datasets exhibit a gradual decrease in difference from crop disease images, with domain similarity values of 0.77/13.53 and 0.81/13.07, respectively. The PlantVillage, Flower, and Pest datasets, characterized by their consistent semantic content and complex backgrounds, belonged to the source domain datasets with high visual similarity. Among these, the Pest dataset exhibits the closest data distribution to the target domain, boasting a domain similarity of 0.93/7.46.

**Table 2:** Measurement of similarity between the source and target datasets.

Similarity metrics	Cars	Leaves	CUB200	Plant Village	Flower	Pest
Cosine similarity	0.65	0.77	0.81	0.85	0.88	0.93
Euclidean distance	17.13	13.53	13.07	12.21	9.95	7.46

The cross-domain performance of three CD-FSL models for disease identification was assessed using six datasets as source domains and FCDD as the target domain. As shown in **Table 3**, the classification accuracy of the three CDFSL methods significantly increased with increasing domain similarity in the 5-way 5-shot case. Among these source domains, CDFSL-MAML, CDFSL-BDC and CDFSL-NML perform best in the high similarity Pest dataset, with their accuracies of 59.94%, 80.13% and 83.61%, respectively. Compared to the low-similarity Cars dataset, the accuracy of these three methods improves by 21.01%, 6.52% and 20.44%, respectively. Similar trends are observed in the 5-way 1-shot configuration (**Table 4**). However, the accuracy shows a substantial decrease on all source domains. For the Pest source domain, the accuracies of the three CD-FSL methods in the 5-way 1-shot configuration are only 42.63%, 63.95% and 66.82%, respectively, which are 17.31%, 16.48%, and 16.79% lower than in the 5-way 5-shot configuration. The experimental results indicate that the inter-domain distribution differences have a large impact on the generalization performance of CD-FSL. It is noteworthy that while the semantic information of the PlantVillage dataset aligns closely with the target domain and encompasses most disease types in FCDD, its accuracy trailed behind Flowers and Pest in both the 5-way 1-shot and 5-way 5-shot scenarios. One possible reason for this is that the background of the PlantVillage data remains fixed, while FCDD exhibits diverse backgrounds. The CD-FSL models may struggle to learn background variability on the PlantVillage dataset during meta-training. In addition, we observed consistent domain similarity intervals for the data from the three source domains, Flower, Pest, and PlantVillage. However, the CD-FSL model performed better on the Pest dataset than the other datasets. The main reason for this phenomenon is that the Pest dataset contains plants and environmental conditions similar to the field disease images, which provides a more representative training sample for the models. This suggests that increasing the complexity of the data (e.g., diverse plant species and growing environments) can significantly improve the cross-domain generalisation ability of the model in the context of the same domain similarity. In future research, we will explore the integration of adversarial training, domain adaptation techniques, and multi-domain co-training strategies within the framework of FSL to effectively deal with the FSL disease classification problem under domain shifting.

**Table 3:** Disease recognition accuracy of 5way-5shot setting in different cross domain scenarios.

Methods	Cars→ FCDD	Leaves→ FCDD	CUB200→ FCDD	PlantVillage→ FCDD	Flower→ FCDD	Pest→ FCDD
CDFSL-MAML	38.93	39.38	41.93	54.16	48.29	59.94
CDFSL-BDC	73.61	72.99	73.82	77.42	78.94	80.13
CDFSL-NML	63.17	66.54	69.74	75.81	75.41	83.61

**Table 4:** Disease recognition accuracy of 5way-1 shot setting in different cross domain scenarios.

Methods	Cars→ FCDD	Leaves→ FCDD	CUB200→ FCDD	PlantVillage→ FCDD	Flower→ FCDD	Pest→ FCDD
CDFSL-MAML	31.43	32.98	33.94	40.28	37.41	42.63
CDFSL-BDC	52.45	52.98	55.28	62.46	62.42	63.95
CDFSL-NML	47.11	49.68	52.01	58.27	59.16	66.82

Overall, CDFSL-BDC and CDFSL-NML show remarkable performance gains in cross-domain learning. Conversely, CDFSL-MAML exhibits comparatively poor performance for cross-domain disease identification. Specifically, for the 5-way 5-shot task on the Pest dataset, CDFSL-MAML achieves an accuracy of only 59.94%. Similarly, its accuracy for the 5-way 1-shot task is also relatively low. These comparison findings suggest that the optimization-based meta-learning method (CDFSL-MAML) struggles to generalize meta-learning knowledge to unseen categories when the source and target domains have large domain gap. Additionally, the accuracy of the non-meta-learning approach (CDFSL-NML) exceeds that of the meta-learning approach (CDFSL-BDC) only on the source domain Pest. This is because CDFSL-NML, unlike task-driven meta-learning methods, employs a non-episodic training mode on the source domain datasets. Therefore, if this method aims to achieve better recognition accuracy in CD-FSL task, it requires a dataset with high domain similarity as the source domain. This result has a similar conclusion to the traditional transfer learning plus fine-tuning method.

Another observation shows that the performance of all models varies significantly across different similarity domains. In particular, CDFSL-BDC shows significant competitiveness on low similarity datasets such as Cars, Leaves, and CUB200. Specifically, for the 5way-5shot task, this method achieves accuracy improvements of 10.44%, 6.45%, and 4.08% on these three datasets compared to CDFSL-NML. In low-similarity domains, the generalisation ability of the model is limited due to the large feature distribution differences between the source and target domains, leading to its poor performance in such domains. This phenomenon is particularly prominent in CDFSL-MAML, mainly due to the diversity and complexity of agricultural environments, which prevents generic initialisation models from effectively adapting to task-specific features, thus affecting their adaptability and performance in new domains. In addition, CDFSL-BDC outperforms CDFSL-NML and significantly outperforms CDFSL-MAML on the high similarity datasets PlantVillage and Flower. In these high similarity domains, the feature distributions between the training data and the test data are more consistent, so the model is able to extract effective information from the limited samples and perform inference more easily, thus better migrating the existing knowledge and improving the inference effect. In the 5-way-1-shot task, CDFSL-BDC achieves the best average performance, further validating its advantages in high similarity domains. This result indicates that the model is able to learn and adapt more efficiently in high similarity contexts with smaller data volumes, reflecting its strong domain adaptation and knowledge transfer capabilities.

Overall, we found that the accuracy differences mainly stem from the differences in high-dimensional feature embedding, learning approaches, and network structures among the three models. Specifically, the CDFSL-BDC model effectively mitigates the problem of inter-domain differences in CD-FSL and enhances the cross-domain migration capability by embedding the BDC module and episodic training strategy, especially on low similarity data. Although CDFSL-NML also achieves comparable accuracy to CDFSL-BDC, its performance is easily limited by data distribution. It should be emphasised that CDFSL-MAML mainly relies on meta-learning methods, which are less adaptive to inter-domain differences, especially in cross-domain tasks where inter-domain feature differences may lead to a model that does not perform as well as expected in a new domain.

### 3.3 Effect of different universal feature extractors on the cross-domain performance

To validate the effectiveness of the universal feature extractor CBAM+ResNet on different inter-domain similarity datasets, confusion experiments were conducted on the source domain data Cars and Pest (**Table 5**). Cars and Pest represent low and high similarity datasets, respectively. After the integration of CBAM module into the baseline networks ResNet-18 and ResNet-12, the accuracy of the three CD-FSL methods on the FCDD target domain shows significant improvement. Furthermore, compared to the Pest data, the improved universal feature extractor performs better on the Cars dataset with larger domain differences. Specifically, on the Cars and Pest datasets, CDFSL-BDC increased by 3.57% and 1.79%, CDFSL-NML increased by 3.69% and 2.37%, and CDFSL-MAML increased by 3.08% and 1.99% respectively. These results demonstrate that the CBAM module enhances the general feature representation capability of the CD-FSL model and has a positive effect on cross-domain generalization.

**Table 5:** Confusion experiments of universal feature extractor.

Feature extractor	CD-FSL method	Source domain	5way-5shot
ResNet-18	CDFSL-BDC	Cars	70.04
ResNet-18+CBAM	CDFSL-BDC	Cars	73.61
ResNet-18	CDFSL-BDC	Pest	78.34
ResNet-18+CBAM	CDFSL-BDC	Pest	80.13
ResNet-18	CDFSL-NML	Cars	59.48
ResNet-18+CBAM	CDFSL-NML	Cars	63.17
ResNet-18	CDFSL-NML	Pest	81.24
ResNet-18+CBAM	CDFSL-NML	Pest	83.61
ResNet-12	CDFSL-MAML	Cars	35.85
ResNet-12+CBAM	CDFSL-MAML	Cars	38.93
ResNet-12	CDFSL-MAML	Pest	57.95
ResNet-12+CBAM	CDFSL-MAML	Pest	59.94

### 3.4 Comparison with related methods

**Figure 9:**
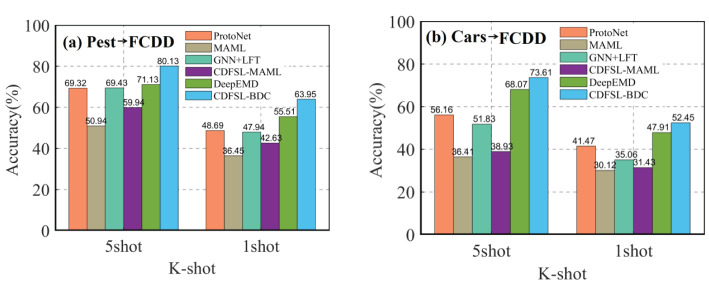
Cross-domain comparison of our CD-FSL models with relevant methods in both 5-shot and 1-shot settings. **(A)** Pest to FCDD. **(B)** Cars to FCDD.

The proposed CDFSL-BDC and CDFSL-MAML were compared with several popular FSL methods, including ProtoNet (Snell et al., 2017), MAML (Finn et al., 2017), GNN+LFT (Tseng et al., 2020), and DeepEMD (Zhang et al., 2020). As can be seen in **Figure 9**, the CDFSL-BDC model achieves the best accuracy in both the Cars→FCDD and Pest→FCDD settings. In all cross-domain scenarios, while the accuracy of the CDFSL-MAML model is superior to that of the MAML model, its performance is significantly lower than that of the comparison methods. This result further indicates that optimization-based meta-learning methods lose their advantage in cross-domain learning of diseases, a conclusion that is consistent with previous research results (Chen et al., 2019). Given that the CDFSL-BDC model was developed based on ProtoNet, we compare our model with the ProtoNet model. Specifically, under the Pest→FCDD 5-shot and 1-shot settings, the accuracy of the CDFSL-BDC is improved by 10.81% and 15.26% respectively compared to ProtoNet. Under the Cars→FCDD cross-domain, the accuracy is improved by17.45% and 10.98%. This result indicates that the introduction of the general feature extractor CBAM module and BCD module can effectively improve the cross-domain learning ability of FSL.

### 3.5 Cross-domain performance evaluation under multi-domain learning

**Figure 10:**
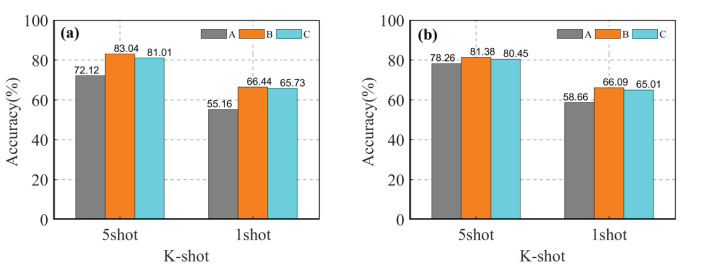
Compare average accuracy of CDFSL-NML **(A)** and **(B)** CDFSL-BDC under multi-domain learning.

In the multi-domain learning, during meta training, (*S*, *Q*) tasks were sampled from multiple datasets. The source domain data is combined into three multi-domain datasets, namely A, B, and C, based on their inter-domain differences. The purpose of this experiment is to investigate how multi-domain learning affects the recognition accuracy of CD-FSL. The datasets are as follows: low-similarity dataset A={Cars, Leaves, CUB200}, high-similarity dataset B={PlantVillage, Flower, Pest} and mixed dataset C = {Cars, Leaves, CUB200, PlantVillage, Flower, Pest}. The specific procedure is to extract all the categories of the different domains to reintegrate them into a new source domain data. In meta-training, the merged multi-domain dataset is utilised as an overall source domain to train the CD-FSL model in order to reduce the complexity of model training. The same network model and parameter settings are used to train the CD-FSL model using A, B, and C source domain data. According to **Figure 10**, the accuracies of the two CD-FSL methods show significant improvement when the multi-domain A is used as the source domain, compared to the single domain. In the 5-shot case, CDFSL-NML improves the accuracies by 8.95%, 5.58% and 2.38% on multi-domain A compared to single-domain Cars, Leaves, and CUB200, respectively. For the 1-shot case, CDFSL-NML improves the accuracies by 8.05%, 5.48% and 3.15% on multi-domain A, respectively. Similarly, CDFSL-BDC, trained on multi-domain A, outperforms each single-domain learning. This result suggests that multi-domain learning can significantly improve the cross-domain generalisation ability of FSL, especially in cases with large differences between domains. Except for Pest dataset, the accuracy of CD-FSL learned on multi-domain B is higher than that of single-domain PlantVillage and Flower. Although multi-domain C has a large image capacity, large number of categories and rich knowledge, the recognition accuracy has not been greatly improved. On the contrary, multi-domain B obtains better results than multi-domain C on 5shot and 1shot. In addition, the comparison found that the performance of multi-domain A, B and C on 5shot and 1shot never exceeded that of single-domain Pest. This result shows that reducing inter-domain differences is more effective than increasing domain diversity in cross-domain FSL.

### 3.6 CD-FSL for single-crop disease identification

Fine-grained disease classification is of greater significance for practical applications, but is also a challenging task. This is because different disease types within the same crop may exhibit similar visual features. By evaluating the performance of CD-FSL on different crops, we can gain a more comprehensive understanding of the model’s generalization ability and adaptability in different environments. The cross-domain performance of CD-FSL was tested on nine different crop disease datasets using the Pest dataset as the source domain. As shown in **Table 6**, the accuracy of both CDFSL methods under a single crop dataset is significantly lower compared to the test results under FCDD. Therefore, CD-FSL has more difficulties in identifying disease types of single crops. This is because the granularity of single-crop datasets is finer than FCDD, and the distribution of visual features between different diseases may be similar. Similarly, the accuracy of CDFSL-NML was better than that of CDFSL-BDC under the single crop disease dataset, which is consistent with the previous conclusion. Overall, with the exception of tomato and apple crops, CD-FSL achieved satisfactory results on challenging crops. On the potato and grape datasets, the accuracy of CDFSL-NML is comparable to the results on the FCDD dataset. The experimental results show that CD-FSL has a strong robustness and domain adaptive ability to adapt to a variety of different crops and disease types. In addition, the experimental observations also revealed that there is a significant difference in the accuracy of CD-FSL for different crop diseases. This is due to the fact that the FCDD dataset was captured by different sensors, backgrounds, light and noise conditions, resulting in varying levels of background complexity among different samples.

**Table 6:** Comparison of recognition accuracy for different crop categories.

ID	Crop types	N-Way	Number of categories	CDFSL-BDC		CDFSL-NML	
				5shot	1shot	5shot	1shot
1	Potato	3way	3	76.48	58.95	81.02	62.55
2	Corn	4way	4	60.67	45.38	64.74	52.73
3	Cotton	5way	7	70.50	50.63	75.20	54.89
4	Rice	4way	4	67.71	47.69	72.79	55.49
5	Grape	4way	4	74.21	57.92	85.75	65.83
6	Tomato	5way	10	56.54	45.51	59.38	47.36
7	Wheat	3way	3	69.89	55.05	73.16	58.26
8	Apple	4way	4	53.63	40.47	57.47	41.46
9	Cucumber	4way	4	69.95	47.91	76.63	56.61

**Figure 11:**
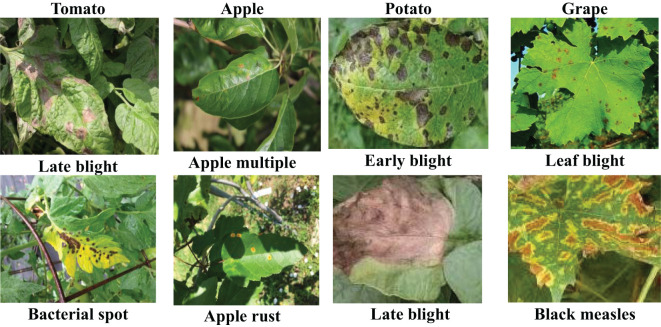
Visual comparison of difficult disease images with easily recognisable images. The apple and tomato categories are difficult disease images. All these sample images have complex backgrounds. The potato and grape categories fall into the category of easily recognisable disease images.

Potato and grape crops have 3 and 4 disease types respectively. As shown in **Figure 11**, the diseased leaves occupy most of the image area in the image, and all the symptoms have very obvious characteristics. Therefore, CD-FSL performs better on these two crop datasets. In contrast, in the tomato and apple examples, the image background area is extremely complex, occupying a substantial portion of the image. Observations also show that the diseased areas of the apple crops are smaller and the symptoms are very similar. Therefore, both CDFSL-BDC and CDFSL-NML struggle to effectively identify diseases in apples and tomatoes. This challenge arises because a significant portion of the variation between the support set and query set samples in the meta-test lies within the background, and significant background disturbances diminish the generalization ability of the model.

**Figure 12:**
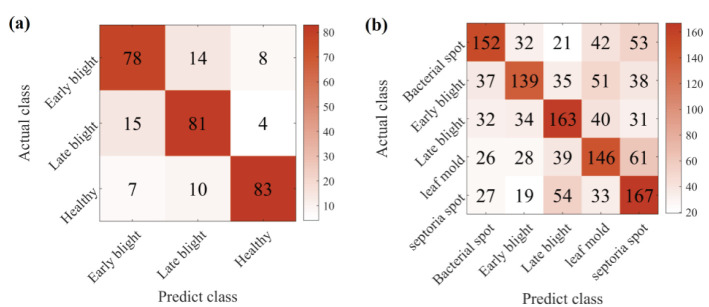
Tomato and potato classification results by CDFSL-BDC model. **(A)** Potato. **(B)** Tomato.

We further analyse the classification performance of CDFSL-BDC for each disease type in potato and tomato. These two crops represent contexts of different complexity. **Figure 12** shows the cumulative confusion matrix under 5shot. As can be seen in **Figure 12A**, CDFSL-BDC accurately identified most of the potato early and late blight sample images. Similarly, the model also performed well in distinguishing potato health from other disease categories. However, the CDFSL-BDC model could not distinguish the disease types of tomato well. The recognition accuracy range from 46.33% to 55.67%. This indicates that the complex background is still a major factor in the recognition accuracy of image diseases.

## 4 Discussions

### 4.1 Effect of source domain size on disease recognition accuracy

**Figure 13:**
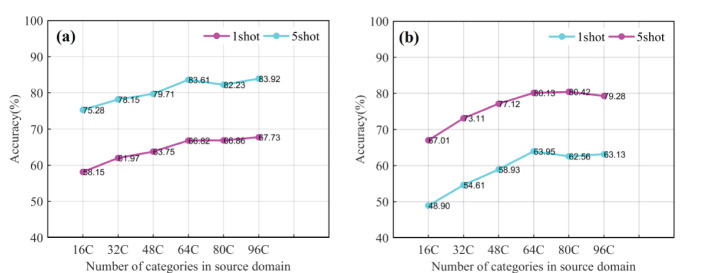
Comparison of two CD-FSL models in terms of disease identification accuracy under different source domain data categories. **(A)** CDFSL-NML, **(B)** CDFSL-BDC.

Large source domain datasets can provide more comprehensive information for models, but excessively large data sizes can result in increased computational overhead. To further explore how the size of the source domain data affects CD-FSL performance, we created six different sizes of source domain data using the Pest dataset. Subsequently, CDFSL-NML and CDFSL-BDC were retrained with the sub-source domain data, followed by meta-testing in FCDD for both 5-shot and 1-shot tasks. As shown in **Figure 13**, the accuracy of both CD-FSL models significantly improves as the number of source domain categories increases, due to the enriched knowledge provided by a larger variety of source domain categories. However, the generalisation performance of the models stabilises when the number of source domain categories exceeds 64. This result suggests that larger source domain data sizes do not necessarily yield better results. Compared to the 1-shot task, increasing the number of categories in the source domain is more effective in improving the recognition accuracy of the 5-shot task. Specifically, when the number of categories is increased from 16C to 64C, the accuracy of CDFSL-NML and CDFSL-BDC is improved by 8.33% and 13.12%, respectively. In addition, another observation shows that CDFSL-BDC is more sensitive to changes in the number of source domain categories. The maximum accuracy change reached 13.41% and 15.05% in the 5-shot and 1-shot tasks, respectively, both higher than the maximum change in CDFSL-NML. One possible reason is that CDFSL-BDC adopts a meta-learning strategy, focusing on how the model learns rather than directly learning the knowledge itself. The limited number of source domain categories may lead to a serious lack of learning capability of the model. The experimental results show that the size of the source domain indeed plays a key role in cross-domain learning tasks, especially in meta-training. Larger source domains can provide more samples and richer feature information, thus helping the model to generalise better to the target task. In practice, when the difference between domains is small, the number of source domain samples is reduced appropriately, based on ensuring the diversity of the source domains. Specifically, different weights are assigned to the data in different domains according to the similarity between the tasks in the source domain and the target domain. In this way, the capacity of the source domain can be selected based on its similarity to the target domain, thus maximising the generalisation performance and reducing unnecessary computational overhead.

### 4.2 Motivation, contribution of the study

Acquiring a large amount of labeled disease images can be an expensive and time-consuming task for disease recognition. Therefore, there is an urgent need to develop a cross-domain machine learning model suitable for limited labeled data. Although FSL provide a promising approach to address data scarcity issues, typical FSL models perform poorly when applied to the field setting. In addition, most FSL methods have a restrictive setup for their application scope, where training and testing samples come from a single domain (or data distribution) (Li and Chao, 2021). For instance, some studies have used some categories from the PlantVillage dataset as base classes and the remaining categories as novel classes. This severely limits the usefulness of FSL in disease applications.

In this work, we attempt to explore a CD-FSL disease recognition approach from a data perspective, aiming to extend disease recognition from the laboratory to field scenarios. The results show that given an arbitrary domain, cross-domain learning can identify new categories from unknown disease The three CD-FSL models show their advantages in different application environments. The CDFSL-BDC model has more potential in complex field environments with large inter-domain differences. When dealing with high similarity datasets (e.g., Pest), CDFSL-NML can achieve high recognition accuracy. Despite the lower accuracy of CDFSL-MAML in such tasks, its ability to quickly adapt to a small number of samples makes it still useful in simplified scenarios or rough estimation tasks. In addition to models and algorithms, datasets are also a central element to improve the accuracy of disease recognition. Therefore, we first collected samples from publicly available resources and large field fields to build a more realistic and closer to actual farm conditions crop disease image dataset for subsequent few-shot classification. Second, we explored the effect mechanism of cross-domain and multi-domain learning on disease identification, which will provide optimization solutions for designing crop disease identification models. Overall, cross-domain FSL can address the issues of data scarcity, domain differences, knowledge sharing and generalisation capabilities in disease identification. The study of this learning paradigm will contribute to the development of smarter, flexible and adaptable machine learning systems for field crop diseases.

### 4.3 Limitations of the study

In complex backgrounds, images may contain a large amount of background information, such as the texture of plant leaves, light variations, or other environmental features, which can interfere with disease recognition.CD-FSL models often have difficulty in effectively separating disease features from background information, especially when training samples are scarce, and the models have difficulty in learning effective background denoising capabilities from the limited number of annotated samples. Therefore, we propose to use the FSL detection method to detect the diseased region to reduce the influence of background. In some cases, the visual features of disease manifestations may have strong similarities with background objects, especially as some plant diseases are difficult to distinguish from natural ageing of leaves or external injuries in the early stages. Diseases in complex backgrounds may appear at different scales and locations in the image, and the CD-FSL model may face challenges in learning cross-scale features. The morphology of crop disease spots has a significant impact on the recognition performance of the model. Some disease spots may be single, while others may be complex spots caused by multiple pathogens together. Samples with inconspicuous disease symptoms and small spots will be more likely to be misdiagnosed. In fact, to prevent disease transmission to other plants, farmers are more likely to need intelligent plant protection robots that can accurately diagnose the type of crop disease at its earliest stage of development. In addition, the performance of CD-FSL is still far from satisfactory when the distribution of different domains varies greatly. We expect that models trained on arbitrary data can be applied to disease identification without the need to collect samples similar to the target domain. This will greatly reduce the cost of applying deep learning models to the disease domain. Future research could explore the integration of different data modalities (e.g., images, sensor data, meteorological data, etc.) to provide richer information sources to help the model better adapt to complex backgrounds and variable environments. In order to improve the recognition ability of CD-FSL in complex backgrounds, future research can focus on improving feature extraction techniques. For example, more advanced CNN architectures, attention mechanisms, or self-supervised learning methods can be used to enhance the model’s ability to recognise subtle disease features.

## 5 Conclusion

Automatic identification of plant diseases plays a pivotal role in advancing agricultural informatization and intelligence. The challenge of improving the accuracy of plant disease identification using deep learning technology is not only related to models and algorithms, but also to the limited availability of disease data. Therefore, the utilization of FSL methods, particularly cross-domain FSL, holds immense promise in addressing the paucity of crop disease samples. This study proposes a broader cross-domain FSL framework for disease recognition tasks in field scenarios, based on an improved universal feature extractor, and explores the differences between three CD-FSL methods in cross-domain learning. Additionally, a comprehensive crop disease dataset, FCCD, is constructed to evaluate the adaptability of the CD-FSL model to new tasks. Experimental results demonstrate that reducing inter-domain differences can significantly improve the recognition accuracy of all cross-domain FSL models. For the 5shot/1shot task, CDFSL-NML, CDFSL-BDC, and CDFSL-MAML achieve recognition accuracies of 83.61%/66.82%, 80.13%/63.95% and 59.94%/42.63% on the Pest benchmark data, respectively. Compared to other relevant FSL methods, CDFSL-BDC shows significant competitiveness in cross-domain generalization. Multi-domain learning improves the knowledge transfer capability of all FSL models, but increasing data diversity is less important when the source domain uses datasets with high similarity metrics. Overall, considering inter-domain correlation and optimizing universal feature extractors in CD-FSL can significantly improve cross-domain disease recognition accuracy.

In this work, we attempt to reveal the impact of inter-domain differences and the diversity of source domain data on CD-FSL performance from a data perspective. Although CD-FSL has made promising progress in addressing the challenging nature of disease scarcity, its performance is still far from satisfactory when inter-domain differences are large or when the background of disease images is too complex. On the other hand, generic CD-FSL models tend to focus on a few biased and simple visual features, and these unimodal features may not provide advanced semantic information for cross-domain generalisation. Possible remedies include the addition of proposing smarter multi-domain FSL or multimodal learning methods to integrate the extracted multimodal knowledge into the target task in a more efficient fusion way. The future application of the CD-FSL model is promising. By introducing an adaptive mechanism, the model can be extended to crops with higher diversity and complex disease types, while adapting to diverse environmental conditions, such as greenhouse cultivation, open field, and complex climatic regions. In addition, by combining multimodal data and time-series information, the model is expected to achieve dynamic disease prediction and precision control, and provide low-cost and high-efficiency solutions for agricultural diseases in resource-poor regions.

## Data Availability

The raw data supporting the conclusions of this article will be made available by the authors, without undue reservation.
